# When Criticism Fails: An Answer to Kelly (2026) on Debunking *When Prophecy Fails*

**DOI:** 10.1002/jhbs.70076

**Published:** 2026-10-13

**Authors:** Stéphanie Baggio, Rémy Amouroux

**Affiliations:** ^1^ Institute of Psychology University of Lausanne Lausanne Switzerland; ^2^ Institute of Primary Health Care (BIHAM) University of Bern Bern Switzerland; ^3^ Institut des Humanités en Médecine (IHM) Centre Hospitalier Universitaire Vaudois Lausanne Switzerland

## Abstract

In his recent article, Kelly (2026) accuses Festinger, Riecken, and Schachter of intentionally misrepresenting their 1956 foundational study, *When Prophecy Fails*. While we acknowledge Kelly's contribution to the literature, we contest the interpretative leap from documenting misrepresentation to alleging intentional misconduct. To illustrate this point, we analysed Kelly's interpretation of Henry Riecken's role, showing how the same archival material can be used to support alternative plausible interpretations. In the absence of clear archival evidence directly supporting the allegation of intentional misconduct, we believe that the move from distortion to misconduct is insufficiently warranted. Our main concern with Kelly's article is therefore that it treats archival materials as if they could support the kind of interpretative closure associated with experimental proof. In doing so, his article risks limiting the very methodological dialogue the psychological humanities seek to foster. A historically reflexive and psychologically informed critique should preserve space for alternative explanations, especially where researchers' theoretical commitments may influence their interpretation of evidence. Allegations of intentional misconduct therefore require strong evidence and serious engagement with such alternatives. In the case of *When Prophecy Fails*, we believe that this standard has not been met.

In *When Prophecy Fails* (1956), Festinger, Riecken & Schachter (1956) presented the results of their covert participant observation of a small millenarian group who expected to be rescued by extraterrestrial visitors from a cataclysmic flood supposed to happen on December 21, 1954. The behavior of the group members after the prophecy failed, namely reaffirmation of their beliefs and proselyting, was interpreted by Festinger et al. as confirmation of the theory of cognitive dissonance. The book has since become a canonical study in social psychology.

Drawing on recently unsealed archival material in the Leon Festinger Papers, Kelly (2026) argues that *When Prophecy Fails* misrepresented the findings and stating that some of the book's central claims are false. According to his interpretation, the group had already been proselytising long before the prophecy failed, after which its members abandoned their beliefs. He also argues that the observers did not merely record the events they described; they actively participated in them. However, Kelly goes further by alleging intentional misconduct on the part of its authors. For example, he states that “the authors knew they were false” (p. 1) and that “the researchers' notes leave no option but to conclude the misrepresentations were intentional” (p. 13). In this response, we challenge this interpretive step, providing concrete examples of previous work in history of psychology and from Festinger et al.'s archival material. Our disagreement with Kelly does not concern scholarly integrity, nor are we seeking to defend the reputation of Festinger et al. Our disagreement concerns what archival sources can establish, particularly whether they can support conclusions about intentions.

We do not dispute that Kelly's broader thesis may ultimately prove to be correct. *When Prophecy Fails* may indeed contain errors, distortions, or biased interpretations. Other scholars have already raised similar concerns (e.g., Dawson 1999; Hardyck and Braden 1962; Stone 2009), and Kelly's use of newly available archival material makes a valuable contribution to this discussion. We would also like to acknowledge Kelly's decision to make redacted digital versions of the archival sources he consulted available via the Open Science Framework. Achieving such transparency is not always easy in historical research, where discussions of open science increasingly focus on Findable, Accessible, Interoperable, and Reusable (FAIR) data, research data management, and the reuse of digitised archival materials, while the circulation of primary sources often remains limited by copyright, privacy, and repository rules (ALLEA 2020). In this case, this is particularly valuable, as it gives readers access to material that would otherwise be difficult to consult and enables other scholars to examine the same documents, consider alternative interpretations, and build upon this archive. What we contest is the interpretive leap from documenting misrepresentation to alleging intentional misconduct. Indeed, misrepresentation does not automatically establish that Festinger and his collaborators knowingly set out to deceive their readers. The history of psychology provides many examples of canonical studies being simplified, mythologised, or retrospectively reconstructed without this necessarily implying deliberate fraud. Researchers with strong theoretical commitments may interpret ambiguous events as supporting their theories without acting in bad faith. This has been well documented in the psychological literature on confirmation bias (Nickerson 1998). Kelly himself acknowledges the prior theoretical commitments of Festinger and his collaborators, which are precisely the conditions under which confirmation bias is likely to operate most powerfully (Nickerson 1998). In the absence of clear archival evidence directly supporting the allegation of intentional misconduct, we believe that the move from distortion to misconduct is insufficiently warranted.

This response emerged from a conversation between a social psychologist, who first read Kelly's article with great interest, and a historian of psychology, whom she then invited to reflect on its implications. This origin is not incidental. It helps explain both the starting point of our discussion and how it developed. The initial reaction, especially the concern with issues such as interpretive transparency, openness, and the need to consider alternative explanations, was shaped by the methodological standards commonly found in contemporary experimental social psychology and its commintment to open science (American Psychological Association 2018). However, this starting point also sparked a productive exchange about the differences between experimental social psychology and the history of psychology as forms of enquiry. The former often seeks relatively decisive inferences from controlled evidence and tends to frame problems in terms of validity, bias, and transparency, while the latter typically works with a greater interpretative latitude, arising not from a choice but from the fragmented nature of its sources, including archives. The main concern with Kelly's article is therefore that it treats archival materials as if they could support the kind of interpretative closure associated with experimental proof. Archives do not work in that way. What is at stake here is not only the possible role of interpretive bias, but also the historiographical status of archival evidence itself. Following Farge and Scott‐Railton (2013), archives should be approached as fragmentary and situated traces rather than transparent repositories of facts. This insight is particularly important here, since archival materials cannot, by themselves, produce the kind of interpretive closure associated with experimental proof. Their use as evidence therefore requires contextualization and caution, especially when alternative interpretations remain plausible. The issue, then, is not whether Kelly is too critical, but whether he closes the interpretation too quickly. In doing so, his article risks limiting the very methodological dialogue that a study of *When Prophecy Fails* could otherwise encourage between experimental social psychology and the history of psychology, that is, the kind of dialogue the psychological humanities seek to foster (Teo 2017; Malich and Rehmann‐Sutter 2022; Freeman 2024).

Another issue worth considering is the relationship between the experimenter and the subject. Danziger (1985) shows that early experimental psychology developed various approaches to this relationship. Some traditions treated subjects as knowledgeable collaborators, while others relied on a clear distinction between researchers and participants. From a different angle, Ash (2015) and Schmid (2011) highlights the ethical implications of the distinction between knowing and unknowing subjects. While concealing the purpose of a study can be methodologically useful, particularly when awareness could alter participants' behaviour, it also conflicts with the principle of informed consent. This tension is directly relevant to *When Prophecy Fails*. The use of covert observation raises legitimate ethical concerns and may also create methodological problems, since the observers themselves influence the situation they seek to observe. However, neither concern amounts to evidence that the researchers deliberately manipulated the situation in order to produce the expected outcome.

More broadly, the history of psychology provides several instructive examples of this kind of critical reappraisal, some of which Kelly himself briefly mentions. Harris's reappraisal of Little Albert showed how a classic case could become a myth in the history of psychology. This myth is shaped not only by Watson's own revisions but also by textbook repetition, theoretical appropriation, and pedagogical simplification (Harris 1979). Manning et al. (2007) reappraisal of the Kitty Genovese case makes a similar point: canonical stories may endure less through empirical evidence than through their power as disciplinary parable, shaping textbook knowledge and the framing of later research. Le Texier (2019) archival reconstruction of the Stanford Prison Experiment is perhaps the closest parallel to Kelly's work, as it directly challenges a canonical account in social psychology by questioning both the accuracy of the familiar narrative and the scientific validity and subsequent influence of the study. However, Le Texier's analysis focuses on mechanisms such as demand characteristics, scripting, incomplete and biased data collection, and the persistence of a simplified narrative, rather than quickly moving towards an allegation of intentional misconduct. Perry's work on Milgram also substantially revised a foundational case, both in her book (Perry 2013) and in later archival reanalyses (Perry et al. 2020). She achieves this by emphasising the heterogeneity of the experimental conditions, the ambiguity of subjects' beliefs, and what she and her coauthors call the “dramaturgical credibility” of the experimental setup. This reopens rather than forecloses interpretation.

Despite their differences in method, genre, and scope, these studies share a form of interpretive prudence: they document distortions, show how canonical narratives are constructed and disseminated, and preserve space for alternative explanations rather than instantly collapsing discrepancy into intentional misconduct. They encourage psychology to reflect on how its canonical findings are produced, presented, taught, and reused. Kelly's article shares similarities with these reappraisals, but it differs from them in the speed with which it turns into allegations about intentional misconduct. The problem lies not in debunking itself, but in a form of debunking that mistakes archival reopening for interpretive closure. Criticism should remain historically reflexive, psychologically informed, and interpretatively humble. To provide a concrete example of Kelly's interpretative closure, we have focused on the case of Henry Riecken.

In Section 4.1, Kelly explained that Riecken had a central role in the group. We agree that the newly unsealed archival material provided convincing evidence that Riecken did indeed occupy a special position. He was known as “Brother Henry” and Mrs Martin often tried to persuade him to take on a leadership role. However, according to the documents, Riecken rarely deviated from the stance of being a “sober‐minded, professional observer[s] who aimed to influence the group as little as possible”, as desired by Festinger and reported by Kelly (p. 6) and there is no archival evidence that Riecken was not inclined to respond positively to leadership requests. The archives contain several examples of Riecken denying that he received or interpreted messages, which suggests that he did not take on a leadership role. This includes the following examples.

Reported by Riecken, during an evening when he was forced to take the lead of the group meeting:

At several points during the evening, Mrs. Martin asked me, did you bring a message for us. I dodged the first time and the second time which occurred during a lull in the medium's transcription, I said, I do not have a message comparable in depth to what she is saying, which I hope was acceptable.

(Riecken 1954a, p. 7)

Reported by Salzman & Bosted:

Henry said that he was not getting direct messages.

(Salzman and Bosted 1954, p. 8–9)

Reported by Riecken, in a conversation between the Tech Sergeant and him:

I sat down with the Tech Sergeant and reread the message with him. Then when he asked me what it meant, I said I didn't know[…].

(Riecken and Festinger 1954, p. 1)

Reported by Riecken, in a conversation between a boy and him:

He said, ‘we understand that you also get writings.’ I said, ‘How do you mean?’ He said, ‘You get messages, don't you? Don't you get writings?’ I said, ‘Do you mean that I get writing like Dorothy does?’ ‘Yes,’ ‘he said.’‘No,’ ‘I said,’ ‘I don't get that’.

(Riecken 1954b, p. 4)

Reported by Riecken, in a conversation between a woman and him:

She asked me to tell her about the flood. I said that it was not my job and that was up to Dr. Laughead or Mrs. Martin. She said, ‘Can't you tell me anything?’ I said, ‘No, this is not my job’.

(Riecken 1954b, p. 14)

Taken together, these examples complicate Kelly's interpretation. They suggest that, although Riecken occupied a special position within the group, he also repeatedly tried to avoid presenting himself as a recipient or interpreter of messages, thus remaining closer than Kelly allows to the stance of being “as passive and objective as circumstances allowed” (Kelly, p. 6). Kelly's account gives too little weight to this contrary evidence.

Another example of Kelly's interpretative stance on Riecken deals with what occurred in the night between December 19 and 20, 1954. Kelly reported that Riecken “mocked Martin's attempts to salvage [the prophety]” (p. 7) and drove what occurred that night, including Martin's despair, Laughead's affirmation of belief, and the Christmas message (p. 8). However, that night's events can be interpreted differently. Regarding how Riecken “mocked” Martin's messages, Riecken wrote the two following notes:

At this point, Dorothy was having another message, and as she wrote it, I could look over her shoulder and see what it was. It was a very confusing message indeed and she made some remark at the end showing it to me to the effect that this must have come from a very high density. I said yeah, that's pretty dense all right and caught Leon staring at me.

(Riecken 1954c, p. 10)

Dorothy had just read an exceptionally obscure message and I had made a rather nasty comment on it.

(Riecken 1954d, p. 1)

After making this comment, Riecken went out and Laughead accompanied him. Although Kelly reads this episode as evidence of intentional misconduct, other interpretations remain possible. For example, it could be seen as a lapse in Riecken's participant observation: he slipped by making a sarcastic remark, then he wanted to get some fresh air but could not go out alone. Then, the “exalted” reaction upon his return could be an attempt to distract from his mistake, or it could reveal his enthusiasm regarding Laughead's perfect display of cognitive dissonance outside, or his inability to remain neutral that night. It is also worth noting that this scene occurred at 4 a.m., a time of day when fatigue could easily have contributed to the slip. This interpretation is consistent with Riecken's own account. He later noted that “*Leon picked up on my irritation and boredom*” (Riecken 1954d, p. 1). This episode clearly shows that Riecken did not remain fully neutral. Yet the same archival material can also be read as documenting a momentary lapse in participant observation, produced by fatigue, irritation, and boredom, rather than intentional manipulation. Finally, we have not found anything that matches Kelly's description: Riecken “rejected the earlier psychic message as inadequate” (p. 8).

Overall, Kelly's analytical approach risks reproducing the very interpretive tendency he attributes to Festinger et al. If the latter appear to have read ambiguous events through the lens of cognitive dissonance, Kelly interprets ambiguous archival materials too quickly through the lens of intentional misconduct. A historically reflexive and psychologically informed critique should preserve space for alternative explanations, especially where researchers' theoretical commitments may influence their interpretation of evidence. Allegations of intentional misconduct therefore require strong evidence and serious engagement with such alternatives. In the case of *When Prophecy Fails*, we believe that this standard has not been met.

## Funding

The authors have nothing to report.

## Ethics Statement

The authors have nothing to report.

## Conflicts of Interest

The authors declare no conflicts of interests.

## Data Availability

The authors have nothing to report.
